# Seasonal malaria chemoprevention effectiveness and drug resistance in Plasmodium falciparum: a matched parallel cohort study, Kenya

**DOI:** 10.2471/BLT.25.294889

**Published:** 2026-09-01

**Authors:** Beatrice Rotich, Emmah Kimachas, Joseph Kipkoech, Erastus Kirwa, Christine Markwalter, David Ekai, Ann Mwangi, Suzanne Van Hulle, Diana Menya, Wendy Prudhomme O’Meara

**Affiliations:** a Academic Model Providing Access to Healthcare, Eldoret, Kenya.; b Duke Global Health Institute, 310 Trent Dr, Durham, North Carolina, NC 27710, United States of America.; c Turkana County Government, Turkana, Kenya.; d School of Medicine, Moi University College of Health Sciences, Eldoret, Kenya.; e Catholic Relief Services, Geneva, Switzerland.; f School of Public Health, Moi University College of Health Sciences, Eldoret, Kenya.

## Abstract

**Objective:**

To evaluate seasonal malaria chemoprevention with sulfadoxine–pyrimethamine and amodiaquine in a semi-nomadic community in north-western Kenya, and to examine the relationship between parasite resistance and breakthrough infections.

**Methods:**

A parallel cohort study conducted during the peak malaria season in 2024 involved 10 randomly selected villages in Turkana Central subcounty, where children younger than 5 years received seasonal malaria chemoprevention, and 10 matched villages in Turkana North subcounty (non-intervention cohort). We conducted surveillance of malaria symptoms weekly. The primary outcome was malaria-like symptoms plus a positive rapid diagnostic test result. We analysed *Plasmodium falciparum* genomic DNA in dried blood spot samples from children with symptomatic infections to identify *pfdhfr* and *pfdhps* gene substitutions associated with sulfadoxine–pyrimethamine resistance.

**Findings:**

Children (198 in the intervention cohort and 200 in the non-intervention cohort) were followed until four weeks after the last chemoprevention cycle. In the intervention cohort, 72% (143/198) completed all five cycles. The incidence of malaria was significantly lower in the intervention than non-intervention cohort (adjusted incidence rate ratio 0.29; 95% confidence interval: 0.13–0.67). *Plasmodium falciparum* genomic DNA analysis of samples from 68 infected children showed that *pfdhps* gene mutations were more common in the intervention than non-intervention cohort and became more frequent as the number of treatment cycles increased.

**Conclusion:**

Seasonal malaria chemoprevention with sulfadoxine–pyrimethamine and amodiaquine was associated with substantial protection against malaria in children in an area where sulfadoxine–pyrimethamine resistance was common. Treated children were more likely to harbour resistant parasites.

## Résumé

**Objectif:**

Évaluer la chimioprévention saisonnière du paludisme à base de sulfadoxine-pyriméthamine et d’amodiaquine au sein d’une communauté semi-nomade du nord-ouest du Kenya et examiner la relation entre la résistance parasitaire et les infections survenues sous traitement.

**Méthodes:**

Une étude de cohortes parallèles menée pendant le pic de la saison paludéenne en 2024 a porté sur 10 villages sélectionnés au hasard dans le sous-comté de Turkana Central, où les enfants de moins de 5 ans ont bénéficié d’une chimioprévention saisonnière contre le paludisme, et sur 10 villages appariés du sous-comté de Turkana Nord (cohorte de contrôle). Nous avons assuré une surveillance hebdomadaire des symptômes du paludisme. Le critère d’évaluation principal était la présence de symptômes de type paludique associée à un résultat positif au test de diagnostic rapide. Nous avons analysé l’ADN génomique de *Plasmodium falciparum* dans des échantillons de sang séché provenant d’enfants présentant des infections symptomatiques afin d’identifier les substitutions des gènes *pfdhfr* et *pfdhps* associées à la résistance à la sulfadoxine-pyriméthamine.

**Résultats:**

Les enfants (198 dans la cohorte d’intervention et 200 dans la cohorte de contrôle) ont fait l’objet d’un suivi jusqu’à quatre semaines après le dernier cycle de chimioprévention. Dans la cohorte d’intervention, 72% d’entre eux (143/198) ont achevé les cinq cycles. L’incidence du paludisme était significativement plus faible dans la cohorte d’intervention que dans la cohorte de contrôle (rapport du taux d’incidence ajusté: 0,29; intervalle de confiance à 95%: 0,13–0,67). L’analyse de l’ADN génomique de *Plasmodium falciparum* sur des échantillons prélevés chez 68 enfants infectés a mis en évidence que les mutations du gène *pfdhps* étaient plus fréquentes dans la cohorte d’intervention que dans la cohorte de contrôle et qu’elles devenaient plus fréquentes à mesure que le nombre de cycles de traitement augmentait.

**Conclusion:**

La chimioprévention saisonnière du paludisme par la sulfadoxine-pyriméthamine et l’amodiaquine était associée à une bonne protection contre le paludisme chez les enfants dans une région où la résistance à la sulfadoxine-pyriméthamine était courante. Les enfants traités étaient davantage susceptibles d’héberger des parasites résistants.

## Resumen

**Objetivo:**

Evaluar la quimioprofilaxis estacional contra la malaria con sulfadoxina–pirimetamina y amodiaquina en una comunidad seminómada del noroeste de Kenia, y estudiar la relación entre la resistencia de los parásitos y las infecciones posvacunación.

**Métodos:**

Durante el período de mayor incidencia de la malaria en el año 2024, se realizó un estudio de cohortes paralelas en el que se incluyó a 10 aldeas elegidas aleatoriamente en el subcondado de Turkana Central (donde los niños menores de 5 años recibieron quimioprofilaxis estacional contra la malaria) y 10 aldeas con características similares del subcondado de Turkana Norte (cohorte sin intervención). Semanalmente llevamos a cabo una vigilancia de los síntomas de malaria. El resultado primario fueron síntomas similares a los de la malaria junto con un resultado positivo en una prueba de diagnóstico rápido. Analizamos el ADN genómico del *Plasmodium falciparum* en muestras de manchas de sangre seca procedentes de niños con infecciones sintomáticas para identificar las sustituciones en los genes *pfdhfr* y *pfdhps* asociadas a la resistencia a la sulfadoxina–pirimetamina.

**Resultados:**

Se hizo un seguimiento de los niños (198 en la cohorte con intervención y 200 en la cohorte sin intervención) hasta cuatro semanas después del último ciclo de quimioprofilaxis. En la cohorte con intervención, el 72% (143/198) de los niños completaron el total de cinco ciclos. La incidencia de la malaria fue significativamente inferior en la cohorte con intervención en comparación con la cohorte sin intervención (cociente de tasas de incidencia ajustada: 0,29; intervalo de confianza del 95%: 0,13–0,67). Los análisis de muestras de ADN genómico de *Plasmodium falciparum* procedentes de 68 niños infectados mostraron que las mutaciones en el gen *pfdhps* fueron más frecuentes en la cohorte con intervención que en la cohorte sin intervención, frecuencia que aumentaba conforme se incrementaba el número de ciclos de tratamiento.

**Conclusión:**

La quimioprofilaxis estacional contra la malaria con sulfadoxina–pirimetamina y amodiaquina se asoció a una protección significativa contra la malaria en niños de una zona en la que era frecuente la resistencia a la sulfadoxina–pirimetamina. Los niños a los que se administró el tratamiento tenían más probabilidades de ser portadores de parásitos resistentes.

## ملخص

**الغرض:**

تقييم الوقاية الكيميائية الموسمية من الملاريا باستخدام سلفادوكسين-بيريميثامين وأمودياكوين في مجتمع شبه بدوي في شمال غرب كينيا، ودراسة العلاقة بين مقاومة الطفيليات والعدوى الاختراقية.

**الطريقة:**

أجريت دراسة أترابية مقارنة بين مجموعتين متوازيتين خلال ذروة موسم الملاريا عام 2024، شملت 10 قرى مختارة عشوائيًا في مقاطعة توركانا الوسطى، حيث تلقى الأطفال دون سن الخامسة وقاية كيميائية موسمية من الملاريا، و10 قرى مماثلة في مقاطعة توركانا الشمالية (مجموعة غير خاضعة للتدخل). أجرينا رصدًا أسبوعيًا لأعراض الملاريا. تمثلت النتيجة الأولية في ظهور أعراض شبيهة بالملاريا بالإضافة إلى نتيجة إيجابية لاختبار التشخيص السريع. قمنا بتحليل الحمض النووي (DNA) الجينومي *لمتصورة المنجلية * في عينات بقع الدم المجففة من الأطفال المصابين بعدوى مصحوبة بأعراض لتحديد استبدالات جينات *pfdhfr * و *pfdhps * المرتبطة بمقاومة السلفادوكسين-بيريميثامين.

**النتائج:**

تمت متابعة الأطفال (198 في مجموعة التدخل و200 في مجموعة عدم التدخل) لمدة أربعة أسابيع بعد آخر دورة من العلاج الكيميائي الوقائي. في مجموعة التدخل، أكمل %72 (143/198) جميع الدورات الخمس. كان معدل الإصابة بالملاريا أقل بكثير في مجموعة التدخل مقارنةً بمجموعة عدم التدخل (نسبة معدل الإصابة المعدلة 0.29؛ فاصل الثقة %95: 0.13 إلى 0.67). إن تحليل الحمض النووي (DNA) الجينومي *لمتصورة المنجلية * لعينات من 68 طفلاً مصاباً، أظهر أن طفرات جين *pfdhps * كانت أكثر شيوعاً في مجموعة التدخل مقارنةً بمجموعة عدم التدخل، وازدادت وتيرتها مع ازدياد عدد دورات العلاج.

**الاستنتاج:**

ارتبط استخدام الوقاية الكيميائية الموسمية من الملاريا باستخدام سلفادوكسين-بيريميثامين وأمودياكوين بحماية كبيرة ضد الملاريا لدى الأطفال في منطقة كانت مقاومة سلفادوكسين-بيريميثامين شائعة. وكان الأطفال الذين تم علاجهم أكثر عرضة للإصابة بالطفيليات المقاومة.

## 摘要

**目的:**

评估在肯尼亚西北部的半游牧社区使用磺胺多辛‑乙胺嘧啶和阿莫地喹进行季节性疟疾化学预防的效果，研究寄生虫耐药性与突破性感染之间的关系。

**方法:**

在 2024 年疟疾高发季进行了一项平行队列研究，在图尔卡纳中部县随机选择 10 个村庄，在这些村庄中，不满 5 岁的儿童接受季节性疟疾化学预防，并另外纳入图尔卡纳北部县的 10 个相匹配村庄（非干预队列）。我们每周开展一次疟疾症状监测。主要结局是疟疾样症状，并且快速诊断检测结果呈阳性。我们分析了出现症状感染儿童的干血斑样本中的*恶性疟原虫*基因组 DNA，识别出与磺胺多辛‑乙胺嘧啶耐药相关的 *pfdhfr*、*pfdhps* 基因位点突变。

**结果:**

我们对儿童（干预队列中的 198 人，非干预队列中的 200 人）进行随访，直至最后一轮化学预防周期结束后 4 周。在干预队列中，72% (143/198) 的儿童完成全部 5 个预防给药周期。干预组疟疾发病率大大低于非干预组（校正发病率比 0.29；95% 置信区间：0.13–0.67）。我们对 68 例感染儿童样本进行了*恶性疟原虫*基因组 DNA 分析，结果表明干预队列的 *pfdhps* 基因突变比非干预队列更加普遍，并会随着预防给药周期次数增加而更加频繁。

**结论:**

使用磺胺多辛‑乙胺嘧啶和阿莫地喹的季节性疟疾化学预防，在磺胺多辛‑乙胺嘧啶耐药普遍流行的地区与儿童获得显著的疟疾防护有很大关系。接受治疗的儿童更有可能携带耐药虫株。

## Резюме

**Цель:**

Оценить эффективность сезонной химиопрофилактики малярии с применением сульфадоксин-пириметамина и амодиахина в полукочевом сообществе на северо-западе Кении, а также изучить взаимосвязь между устойчивостью паразитов к лекарственным препаратам и прорывными инфекциями.

**Методы:**

Параллельное когортное исследование, проведенное в период пика сезона малярии в 2024 году, включало 10 случайно отобранных деревень в подокруге Центральная Туркана, где дети младше 5 лет получали сезонную химиопрофилактику малярии, и 10 сопоставимых деревень в подокруге Северная Туркана (когорта без вмешательства). Авторы еженедельно проводили наблюдение за симптомами малярии. Первичным исходом считалось наличие симптомов, сходных с симптомами малярии, в сочетании с положительным результатом диагностического экспресс-теста. Авторы анализировали геномную ДНК *Plasmodium falciparum* в образцах высушенных пятен крови, полученных от детей с симптоматическими инфекциями, для выявления замен в генах *pfdhfr* и *pfdhps*, связанных с устойчивостью к сульфадоксин-пириметамину.

**Результаты:**

Наблюдение за детьми (198 в когорте вмешательства и 200 в контрольной когорте) продолжалось до четырех недель после последнего цикла химиопрофилактики. В когорте вмешательства 72% детей (143 ребенка из 198) прошли все пять циклов. Заболеваемость малярией была значительно ниже в когорте вмешательства, чем в контрольной когорте (скорректированное отношение показателей заболеваемости 0,29; 95%-й ДИ: 0,13–0,67). Анализ геномной ДНК *Plasmodium falciparum* в образцах, полученных от 68 инфицированных детей, показал, что мутации гена *pfdhps* чаще встречались в когорте вмешательства, чем в контрольной когорте, и учащались по мере увеличения числа циклов лечения.

**Вывод:**

Сезонная химиопрофилактика малярии с применением сульфадоксин-пириметамина и амодиахина была связана со значительной защитой детей от малярии в районе, где устойчивость к сульфадоксин-пириметамину была широко распространена. У детей, получавших лечение, с большей вероятностью обнаруживались устойчивые паразиты.

## Introduction

Seasonal malaria chemoprevention has been used in the Sahel region of Africa for more than a decade to reduce morbidity and mortality in vulnerable populations. In practice, antimalarials are administered to children to protect against malaria infection and disease. The most commonly used regimen is a sulfadoxine–pyrimethamine combination with amodiaquine, which is administered at 28-day intervals throughout the high period for malaria transmission, usually for 3 to 5 months. This drug regimen clears existing infections and the long half-lives of sulfadoxine, pyrimethamine and the primary metabolite of amodiaquine, desethylamodiaquine, ensure there is prolonged protection against new infections.^1^^,^^2^ Randomized trials and large-scale implementation studies have demonstrated that seasonal malaria chemoprevention with this combination is highly effective, safe and cost-effective for preventing malaria disease among children in areas with seasonal malaria transmission.^3^^–^^6^

After the World Health Organization updated guidelines on scaling up seasonal malaria chemoprevention beyond the Sahel region in 2022,^7^ countries in East Africa began exploring the integration of seasonal malaria chemoprevention into their prevention arsenal by targeting areas with highly seasonal transmission. However, parasites in this part of Africa have higher levels of sulfadoxine–pyrimethamine resistance than those in West Africa.^8^^,^^9^ In Kenya, for example, studies have documented the persistence of antifolate resistance, which reduces the parasite’s susceptibility to sulfadoxine–pyrimethamine, despite withdrawal of the combination in the mid-2000s.^8^^,^^10^ Still, there is evidence supporting the continued use of sulfadoxine–pyrimethamine, for example during pregnancy, to prevent malaria in areas with moderate levels of antifolate resistance.^7^^,^^11^^,^^12^ In 2025, a seasonal malaria chemoprevention pilot study in Uganda, where antifolate resistance is high, demonstrated a 90% reduction in malaria cases.^13^ However, the relationship between antifolate resistance and the efficacy of chemoprevention with sulfadoxine–pyrimethamine and amodiaquine is not well understood.

Here we report the findings of the first seasonal malaria chemoprevention programme in an area of Kenya where communities are highly mobile, sleep out in the open and have little access to formal health services, resulting in limited conventional malaria case management and vector control. We used active case detection to determine the incidence of malaria in parallel cohorts of children in intervention and non-intervention communities, and to assess the degree of protection associated with seasonal malaria chemoprevention involving the routine administration of sulfadoxine–pyrimethamine and amodiaquine. In addition, to investigate the relationships between the effectiveness of seasonal malaria chemoprevention, breakthrough infections and parasite resistance, we tested for the presence of genetic mutations associated with antifolate resistance in samples of parasites obtained during incident infections in both cohorts.

## Methods

The study took place in Turkana County in the north-western corner of Kenya, bordering Uganda to the west and Ethiopia and South Sudan to the north. Most areas have limited access to basic infrastructure, such as roads, health facilities and clean water.^14^ The 2019 census reported that 14% of residents were younger than 5 years.^15^ Annually, the mean temperature ranges between 26 and 38 °C and rainfall varies between 120 and 500 mm and is erratic in distribution and timing. Many local families are semi-nomadic and move with their herds of animals in search of pasture because little rain falls each year. Malaria outbreaks are frequent,^16^^,^^17^ with approximately 80% of cases occurring within a span of 5 months and around 30% occurring in children younger than 5 years.

Seasonal malaria chemoprevention was implemented in Turkana Central subcounty during the high transmission season in 2024 by Turkana County’s health ministry with support from the Catholic Relief Services. Treatment was administered in five cycles at 28-day intervals from June to October 2024. The programme targeted children aged 3 to 59 months and more than 40 000 received at least one cycle of chemoprevention.

### Study design

We conducted a prospective, partially randomized, matched, parallel-cohort study to investigate the association between seasonal malaria chemoprevention and the incidence of malaria. Although our intervention was not randomized, study villages were selected randomly to be as representative as possible. In the intervention area (i.e. Turkana Central subcounty), two villages were selected from each of the five wards containing a total of 644 villages (Fig. 1). Within each ward, villages were selected randomly, using samplepps in Stata (Stata, College Station, United States of America) with the probability that a village would be selected proportional to its size. In the non-intervention comparison area (i.e. neighbouring Turkana North subcounty), 10 villages were randomly selected from a total of 47 villages in areas served by four health facilities where malaria test positivity rates were similar to those at facilities serving the selected Turkana Central villages. Subsequently, 20 households were randomly selected in each village by systematic random sampling from complete lists of households.

**Fig. 1:**
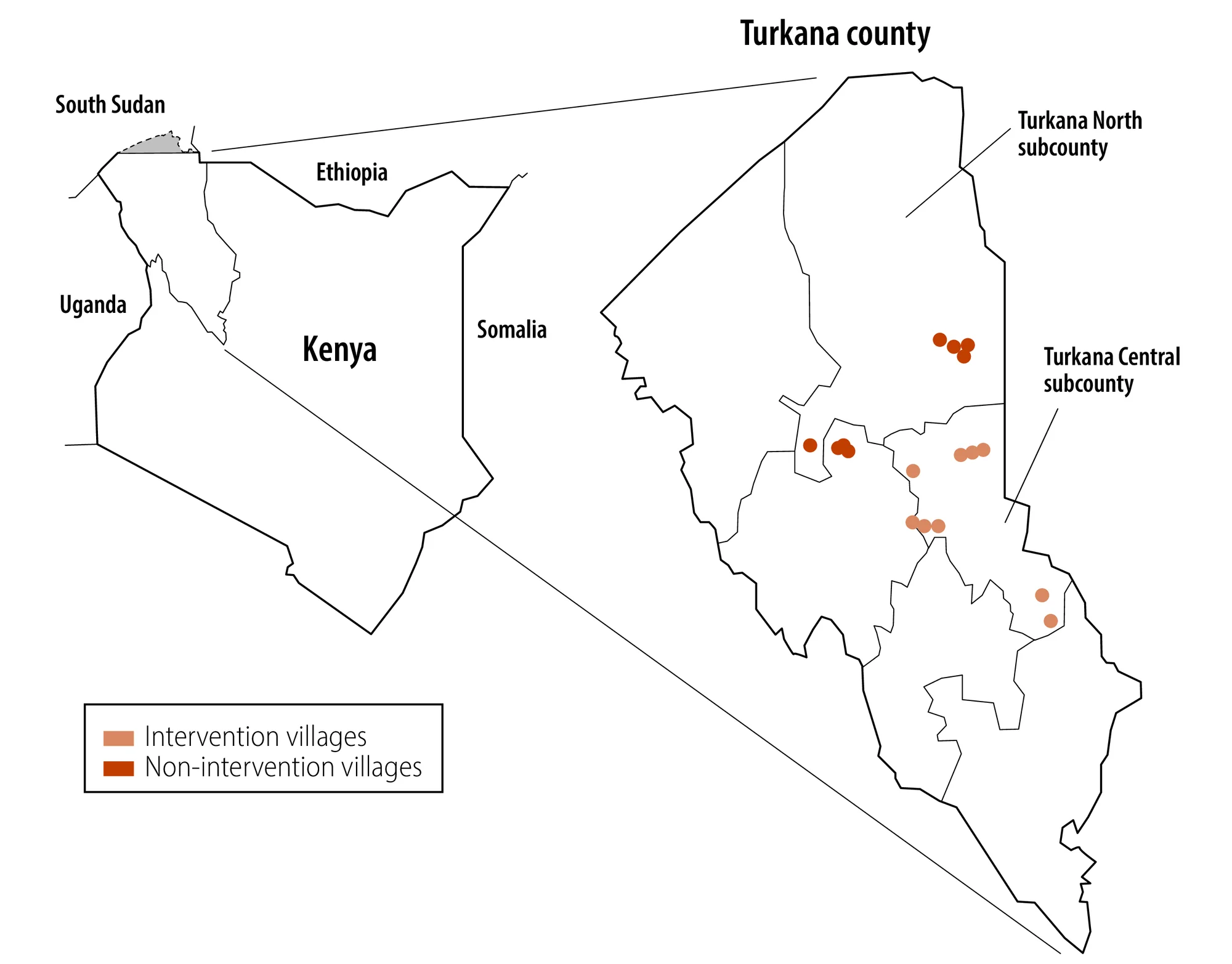
Study villages, Turkana County, study of seasonal malaria chemoprevention and parasite drug resistance, Kenya, 2024

Notes: children in villages marked by brown dots in Turkana Central subcounty received seasonal malaria chemoprevention. Children in villages marked by red dots in Turkana North subcounty served as a non-intervention comparison population. There were 10 villages in each of Turkana Central and Turkana North subcounties but not all are visible on the map because some dots overlap.

A child was eligible for inclusion in the study if they were aged between 6 and 59 months and would not pass their fifth birthday by the start of seasonal malaria chemoprevention in mid-June 2024. If more than one child was eligible in a household, auto-populated, randomized allocation was used for selection. In villages with fewer than 20 households with eligible children, we enrolled additional children from a subset of households.

A sample size of 200 children across 10 clusters in each cohort was sufficient to have 85% power at a 5% level of significance to detect a 50% difference in the proportion of children who had at least one malaria episode. We based our sample size on one episode per child because we lacked prior information about the distribution and likely overdispersion of episodes. This gave us a conservative estimate of the sample size for the desired power and significance. We estimated that 40% of children in the intervention area would have malaria during the 5-month follow-up period.

### Outcome measures

The primary outcome was a positive diagnostic test result for malaria from any source accompanied by malaria-like symptoms, specifically fever, a history of fever, aches, chills and lethargy. The comparison of interest was the incidence of malaria in the cohort receiving seasonal malaria chemoprevention compared to the non-intervention cohort. A secondary outcome was the incidence of any malaria-like illness regardless of whether a test had been performed.

Illnesses were documented through active and passive surveillance. A community health promoter (also known as a community health worker or community health volunteer) visited each household in both intervention and non-intervention areas approximately once per week to ask about fever and illness. When an enrolled child was unwell, the community health promoter offered them a malaria rapid diagnostic test. The community health promoter referred children with a positive test result for treatment at the local health facility, where they received artemether–lumefantrine combination therapy free of charge. In addition, study staff members and community health promoters encouraged households to contact a community health promoter whenever a child had symptoms of malaria to receive a free test. The community health promoters prepared a dried blood spot sample each time they performed a rapid diagnostic test. If a child was unwell and had been taken to a health facility without the community health promoter being contacted, the promoter documented the episode at the next weekly visit. The primary outcome measure included only illnesses accompanied by a positive diagnostic test result. Children were excluded from malaria testing for two weeks following a positive rapid diagnostic test result or malaria treatment to reduce the occurrence of false-positive diagnoses due to circulating antigenaemia. Community health promoters also documented instances when children travelled away from villages.

### Data collection and analysis

At enrolment, we captured household and child data on an electronic form using a REDCap database (Vanderbilt University, Nashville, USA). In addition, community health promoters completed a paper questionnaire on each routine visit and we entered the data collected into the database. Twenty-eight days after the final seasonal malaria chemoprevention cycle, we recorded each child’s participation in the chemoprevention programme in a final survey. We confirmed details by reviewing government-issued seasonal malaria chemoprevention cards, when available.

We conducted an intention-to-treat analysis. The incidence rate ratio of malaria episodes in the intervention group versus the non-intervention group was estimated using a mixed-effects Poisson regression model based on the number of malaria episodes per child. The model was adjusted for prespecified covariates: (i) the sex and age of the child; (ii) mosquito net ownership; (iii) whether the child slept outside; and (iv) the village-level prevalence of malaria infection at baseline. Data were analysed using Stata v. 18 (StataCorp LLC, College Station, USA) and Rstudio v. 2025.05.0+496 (Posit, Boston, USA) with R v. 4.3.1 (The R Foundation, Vienna, Austria), with the following packages: tidyverse v. 2.0.0,^18^ lme4 v. 1.1–34,^19^ survival v. 3.8–3,^20^ survminer v. 0.5.0,^21^ broom v. 1.0.7,^22^ and kableExtra v. 1.4.0^23^

### Laboratory analysis

Dried blood spot samples were prepared from 5 to 10 μL of capillary blood obtained using a finger prick and then adsorbed onto filter paper. We tested samples collected at baseline and on active case detection for the presence of *Plasmodium falciparum* genomic DNA using a nested polymerase chain reaction (PCR) assay, starting with a Phusion Blood Direct PCR Kit (ThermoFisher Scientific, Waltham, USA) with Plu1/5 primers.^24^ A second round of amplification involved previously described primers,^25^ as follows: (i) 1 μL of the primary reaction as template and 0.25 μM of forward and reverse primers; (ii) 0.3 μM probe; and (iii) 1 × SsoAdvanced Universal Probes Supermix (Bio-Rad, Hercules, USA). 

When parasites were present, we extracted fresh *P. falciparum* genomic DNA from dried blood spot samples using a 5-mm punch and a Chelex-based protocol.^26^ In addition, samples underwent testing for genetic markers of resistance to sulfadoxine–pyrimethamine using a PCR allelic discrimination assay.^27^ There are five dominant mutations in the *P. falciparum dhfr* and *dhps* genes that have been shown to correspond to antifolate resistance in East Africa: (i) *pfdhps* A437G; (ii) *pfdhps* K540E; (iii) *pfdhfr* N51I; (iv) *pfdhfr* C59R; and (v) *pfdhfr* S108N. Substitutions *pfdhfr* N51I and *pfdhfr* S108N were at fixation in Kenya (i.e. they were nearly 100% prevalent).^28^ Therefore, we focused our analysis on the remaining three substitutions: *pfdhps* A437G, *pfdhps* K540E and *pfdhfr* C59R. Each PCR reaction included 5 µL of template, 0.3 µM of forward and reverse primers, and 1 × SsoAdvanced Mastermix and 0.2 µM of each of two probes, one corresponding to the mutant allele and the other to the wild-type allele.

### Informed consent

Written informed consent was obtained from the parents or guardians of participating children. Approval was granted by Moi University Institutional Research and Ethics Committee in Kenya and Duke University Institutional Review Board in the United States of America. This study was registered with the Pan African Clinical Trials Registry (PACTR20240570172).

### Participant and public involvement

Participants were not directly involved in the design of the study. However, suggestions and information provided by the public and community leaders during the formative research phase were critical for designing the seasonal malaria chemoprevention programme.^29^ The Kenyan health ministry and community leaders were involved in all aspects of the study design, randomization, enrolment and the supervision of study activities.

## Results

In total, we enrolled 398 eligible children from 389 households in the study: 198 in the intervention cohort and 200 in the non-intervention cohort. Nine households had more than one participating child. The two cohorts were similar at baseline (Table 1). Overall, the children’s mean age was 28.9 months, 49% were female and only 50% had a bednet for their sleeping space. On the day of enrolment, 17% (68/398) reported symptoms and were offered a malaria rapid diagnostic test. Of the 68, 13 (19%) tested positive. Parasite prevalence was measured using a PCR test in all children at enrolment and was found to vary substantially between villages (Fig. 2). Overall, the prevalence was 20% (95% confidence interval, CI: 16–24); it was lower in the non-intervention cohort than the intervention cohort, at 16% versus 23%, respectively (*P* = 0.077). 

**Table 1:** Participants’ characteristics at baseline, study of seasonal malaria chemoprevention and parasite drug resistance, Kenya, 2024

Child’s characteristic	No. (%) of children^a^			*P* ^b^
	Overall (*n* = 398)	Intervention (*n* = 198)	Control (*n* = 200)	
Age in months, mean (SD)	28.9 (14.3)	28.7 (14.5)	29.1 (14.2)	0.77
Female sex	195 (49)	96 (48)	99 (50)	0.84
Has bednet for sleeping space	198 (50)	106 (54)	92 (46)	0.13
Sleeps outside	88 (22)	40 (20)	48 (24)	0.36
Symptomatic at enrolment	68 (17)	38 (19)	30 (15)	0.22
Symptoms plus positive malaria rapid diagnostic test result	13 (3.3)	9 (4.5)	4 (2.0)	0.59

SD: standard deviation.

^a^ All values represent absolute numbers and percentages unless otherwise stated.

^b^ We calculated *P*-values using the *χ^2^* test except for the age comparison, which used a two-sample *t*-test.

Note: Turkana Central was the intervention subcounty, where children received seasonal malaria chemoprevention, and Turkana North subcounty was the control area.

**Fig. 2:**
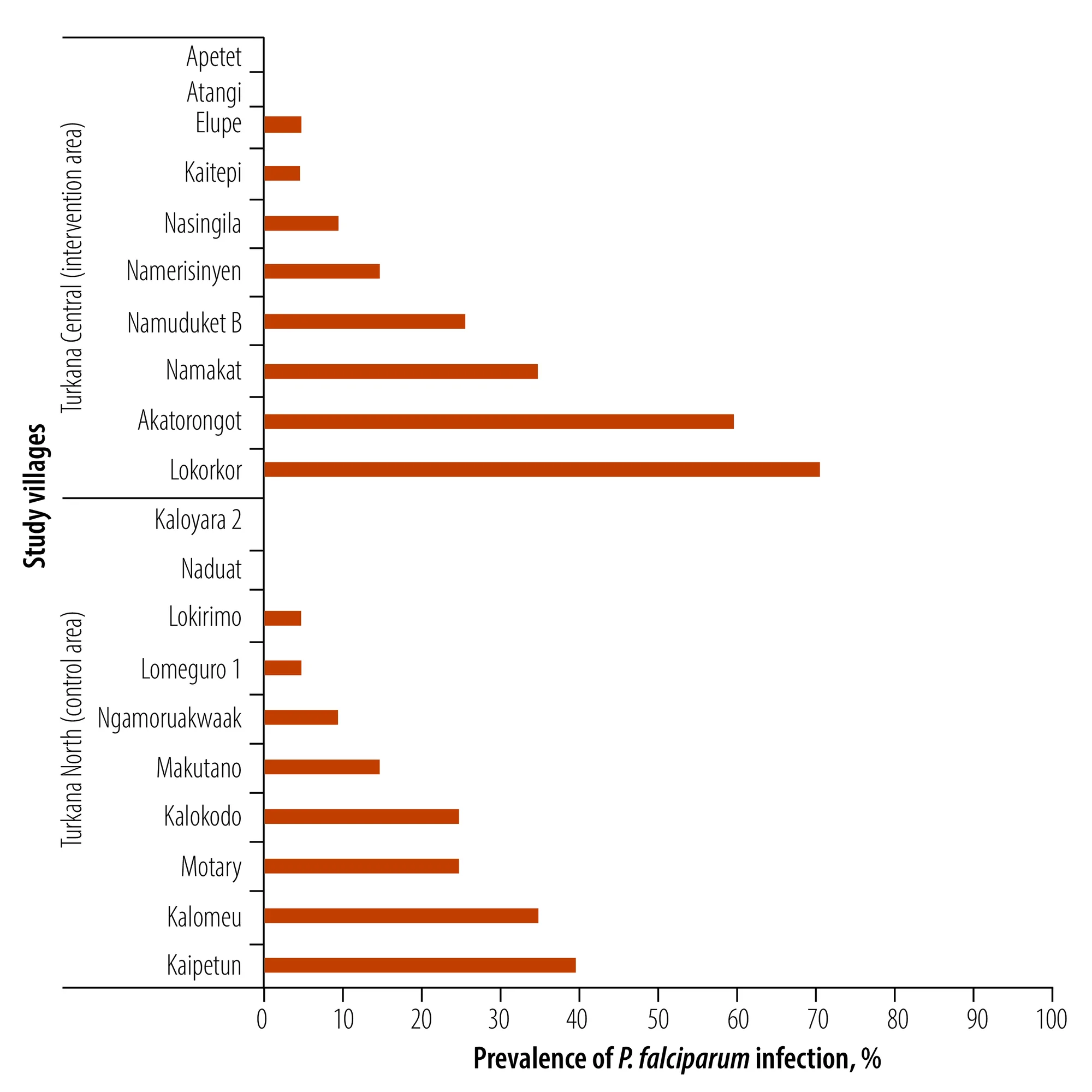
Prevalence of *Plasmodium falciparum* infection at study enrolment, by subcounty and village, study of seasonal malaria chemoprevention and parasite drug resistance, Kenya, 2024

*P. falciparum: Plasmodium falciparum*.

Notes: children in villages in Turkana Central subcounty received seasonal malaria chemoprevention. Children in villages in Turkana North subcounty served as a non-intervention comparison population. The presence of a *Plasmodium falciparum* infection was detected using a polymerase chain reaction (PCR) test.

All 198 children from the intervention area were eligible for seasonal malaria chemoprevention and 143 (72%) received the full five cycles. Of the 55 (28%) who did not receive all five cycles, two (1%) had no chemoprevention at all and 39 (20%) received between one and four cycles. In addition, 14 (7%) children could not be located at the end of the study and their chemoprevention coverage could not be determined fully; however, according to weekly visit records, at least 10 had one or more cycles. 

### Efficacy of seasonal malaria chemoprevention

We recorded malaria episodes from the last day of the first cycle of seasonal malaria chemoprevention administration until 28 days after the last cycle (i.e. from 18 June 2024 to 7 November 2024). Twenty-two children (14 in the intervention cohort and eight in the control cohort) were lost to follow-up and could not be contacted at the end of the study. In addition, 27 children left the study area during the observation period and missed at least two weekly household visits, but 22 of the 27 returned before the final visit. In total, there were 7585 child–weeks of observation: 3779 in the intervention cohort and 3806 in the control cohort. Between the end of the first chemoprevention cycle and 28 days after the last cycle, 566 febrile events were documented, of which 207 were due to malaria: 58 in the intervention cohort and 149 in the control cohort. These figures correspond to a crude incidence of 87.5 malaria episodes per 100 person–years at risk in the intervention cohort and 202.7 per 100 person–years at risk in the control cohort. Overall, 80% (159/198) of children in the intervention cohort remained malaria-free throughout the study period compared to only 50% (99/200) in the control cohort. After adjusting for the children’s age and bednet use and the cluster-level malaria prevalence at baseline, the incidence rate ratio of malaria episodes in the intervention group compared with the non-intervention group was 0.29 (95% CI: 0.13–0.67). The incidence rate ratio of all fevers in the intervention group compared with the non-intervention group was 0.47 (95% CI: 0.31–0.72), which indicates that a high proportion of fevers was due to malaria. Fig. 3 shows the number of malaria cases per week in the intervention and non-intervention cohorts over the study period.

**Fig. 3:**
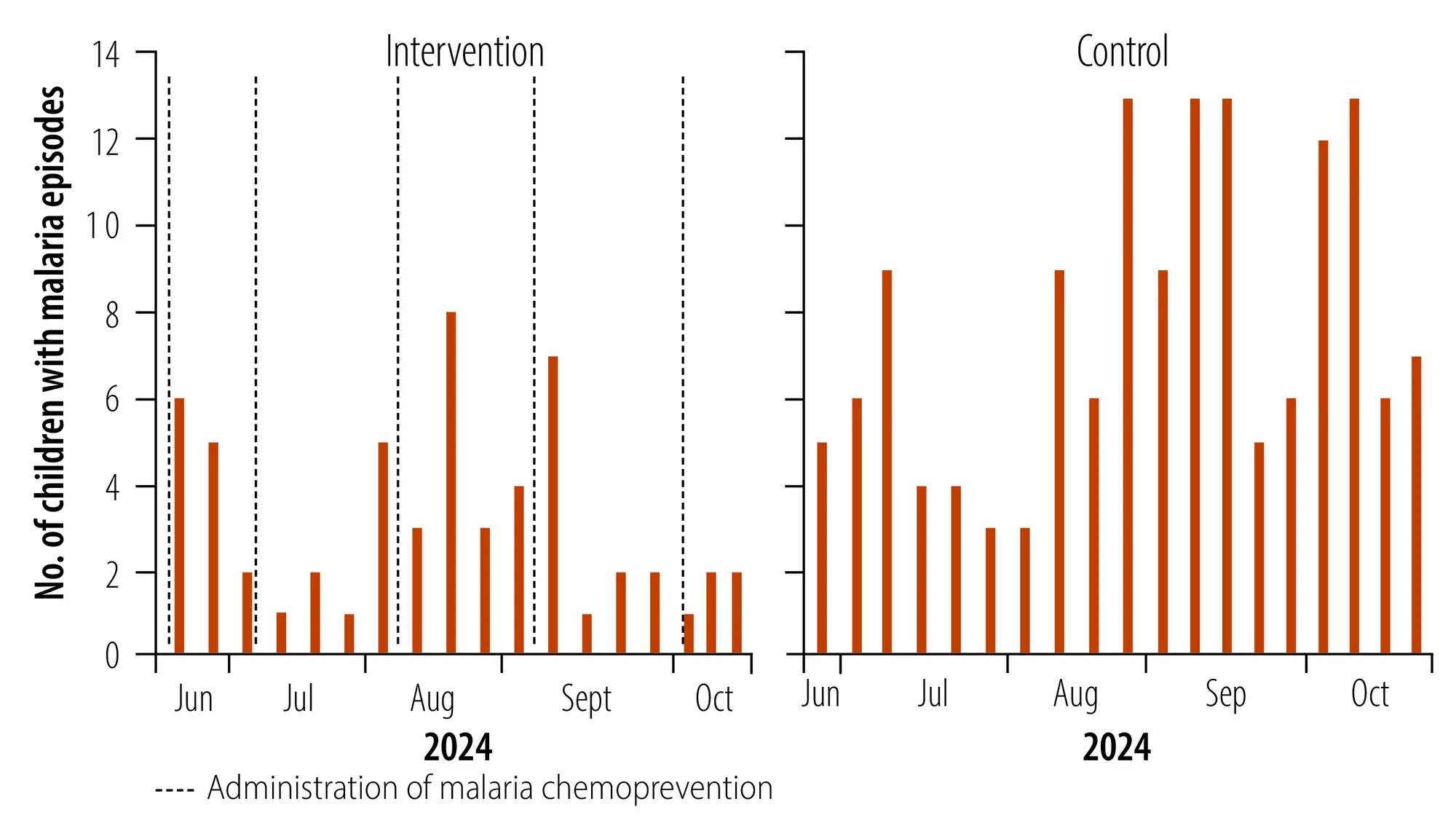
Weekly malaria episodes, by subcounty, study of seasonal malaria chemoprevention and parasite drug resistance, Kenya, 2024

Notes: Turkana Central was the intervention subcounty, where children received seasonal malaria chemoprevention, and Turkana North subcounty was the control area.

Malaria episodes were more common in children who had fewer than five cycles of seasonal malaria chemoprevention than in those who had five cycles; 41 of 58 (71%) malaria episodes observed in the intervention cohort occurred in children with incomplete chemoprevention. Moreover, of the 11 children in the intervention cohort who experienced more than one malaria episode during the study, all but one received fewer than five chemoprevention cycles.

### Parasite resistance

Of the 351 dried blood spot samples collected, 156 contained *P. falciparum*: 55 of 138 (40%) samples from the intervention cohort and 101 of 213 (47%) samples from the control cohort. At least one of the three genomic positions of interest (i.e. *pfdhfr* 59, *pfdhps* 437 or *pfdhps* 540) was amplified in 90 infections and all three positions were amplified in 68. At least one mutation associated with sulfadoxine–pyrimethamine resistance was present in 54% (37/68) of infections for which all three markers of resistance were amplified. The majority of infections contained at least one wild-type allele involving *pfdhfr* 59, *pfdhps* 437 or *pfdhps* 540. More infections in the intervention cohort than the non-intervention cohort had mixed alleles (i.e. both mutant and wild-type alleles; Table 2), which indicates that multi-genomic infections were more common in the intervention cohort. Although the incidence of the *pfdhps* A437G mutation was significantly higher in the intervention cohort than the non-intervention cohort (*P*-value: 0.030; Table 2), the distribution of mutations of interest was not significantly different between the cohorts (*P*-value: 0.063; Table 3). Finally, the odds of harbouring an allele with the *pfdhps* K540E or *pfdhps* A437G mutation increased significantly with each additional seasonal malaria chemoprevention cycle received (Fig. 4).

**Table 2:** Incidence of selected *Plasmodium falciparum* alleles in blood spot samples, by subcounty, study of seasonal malaria chemoprevention and parasite drug resistance, Kenya, 2024

Genomic position and allele type	No. (%) blood spot samples from children with *P. falciparum* infections^a^			
	Overall	Intervention	Control	*P* ^b^
***pfdhfr 59***				
Allele type				0.500
All	86 (100.0)	27 (100.0)	59 (100.0)	NA
Mixed	11 (12.8)	5 (18.5)	6 (10.2)	NA
Mutant	27 (31.4)	7 (25.9)	20 (33.9)	NA
Wild-type	48 (55.8)	15 (55.6)	33 (55.9)	NA
***pfdhps 540***				
Allele type				0.113
All	70 (100.0)	23 (100.0)	47 (100.0)	NA
Mixed	6 (8.6)	4 (17.4)	2 (4.3)	NA
Mutant	12 (17.1)	5 (21.7)	7 (14.9)	NA
Wild	52 (74.3)	14 (60.9)	38 (80.9)	NA
***pfdhps 437***				
Allele type				0.030
All	87 (100.0)	26 (100.0)	61 (100.0)	NA
Mixed	19 (21.8)	7 (26.9)	12 (19.7)	NA
Mutant	12 (13.8)	7 (26.9)	5 (8.2)	NA
Wild	56 (64.4)	12 (46.2)	44 (72.1)	NA

NA: not applicable; *P. falciparum: Plasmodium falciparum*.

^a^ Mutations in *Plasmodium falciparum pfdhfr* and *pfdhps* genes that confer resistance to sulfadoxine–pyrimethamine were detected in dried blood spot samples from children using a polymerase chain reaction (PCR) allelic discrimination assay.

^b^ We calculated *P*-values using the *χ^2^* test for differences in the distribution of the three allele types (i.e. mixed, mutant and wild type) between infections in intervention and control areas.

Note: Turkana Central was the intervention subcounty, where children received seasonal malaria chemoprevention, and Turkana North subcounty was the control area.

**Table 3:** Incidence of sulfadoxine–pyrimethamine-resistance mutations, by subcounty, study of seasonal malaria chemoprevention and parasite drug resistance, northern Kenya, 2024

Resistance mutations detected and level of sulfadoxine–pyrimethamine resistance^a^	No. (%) blood spot samples from children with *P. falciparum* infections			
	Overall (*n* = 74)^b^	Intervention (*n* = 24)	Control (*n* = 50)	*P* ^c^
**Mutations** ^d^				0.063
*pfdhps* A437G alone	2 (2.7)	2 (8.3)	0 (0.0)	NA
*pfdhps* A437G and K540E	6 (8.1)	3 (12.5)	3 (6.0)	NA
*pfdhps* A437G and *pfdhfr* C59R	3 (4.1)	1 (4.2)	2 (4.0)	NA
*pfdhps* A437G and K540E and *pfdhfr* C59R	11 (14.9)	6 (25.0)	5 (10.0)	NA
*pfdhfr* C59R alone	15 (20.3)	2 (8.3)	13 (26.0)	NA
Wild type^e^	37 (50.0)	10 (41.7)	27 (54.0)	NA
**Resistance level^f^**				0.119
0	37 (50.0)	10 (41.7)	27 (54.0)	NA
1	20 (27.0)	5 (20.8)	15 (30.0)	NA
2	17 (23.0)	9 (37.5)	8 (16.0)	NA

NA: not applicable; PCR: polymerase chain reaction; *P. falciparum: Plasmodium falciparum*.

^a^ *P. falciparum* resistance mutations were detected in dried blood spot samples from children using a PCR allelic discrimination assay.

^b^ Samples were obtained from 68 infections in children in whom all three genomic positions of interest (i.e. *pfdhfr* 59, *pfdhps* 437 and *pfdhps* 540) were amplified on PCR analysis plus six infections in children in whom *pfdhps* 540 was not amplified. In these six infections, wild-type *pfdhfr* 59 and *pfdhps* 437 were detected and it was assumed that wild-type *pfdhps* 540 was present because it is very rare for *pfdhps* 540 mutations to occur in the absence of mutations at the other two positions.

^c^ We calculated *P*-values using the *χ^2^* test for differences in the distribution of the six combinations of mutations between infections in intervention and control areas .

^d^ Five dominant mutations in *pfdhfr* and *pfdhps* genes confer resistance to sulfadoxine–pyrimethamine. As previous studies in Kenya found that the prevalence of two of these mutations (i.e. *pfdhfr* S108N and *pfdhfr* N51I) was 100%, which we confirmed in a pilot study involving 10 samples, we assumed all parasites in our study area had these two mutations.

^e^ Wild type indicates that wild-type *pfdhfr* 59, *pfdhps* 540 and *pfdhps* 437 were all detected. For six samples, wild-type *pfdhfr* 59 and *pfdhps* 437 were detected but wild-type *pfdhps* 540 was not, they were also assumed to be wild type.

^f^ The level of sulfadoxine–pyrimethamine resistance among parasites was attributed as follows: (i) 0 for wild-type *pfdhfr* 59, *pfdhps* 540 and *pfdhps* 437; (ii) 1 (i.e. low) for mutations at *pfdhps* 437 alone, *pfdhfr* 59 alone, and *pfdhps* 437 plus *pfdhfr* 59; and (iii) 2 (i.e. high) for mutations at *pfdhps* K540E, with other mutations or alone.

Note: Turkana Central was the intervention subcounty, where children received seasonal malaria chemoprevention, and Turkana North subcounty was the control area.

**Fig. 4:**
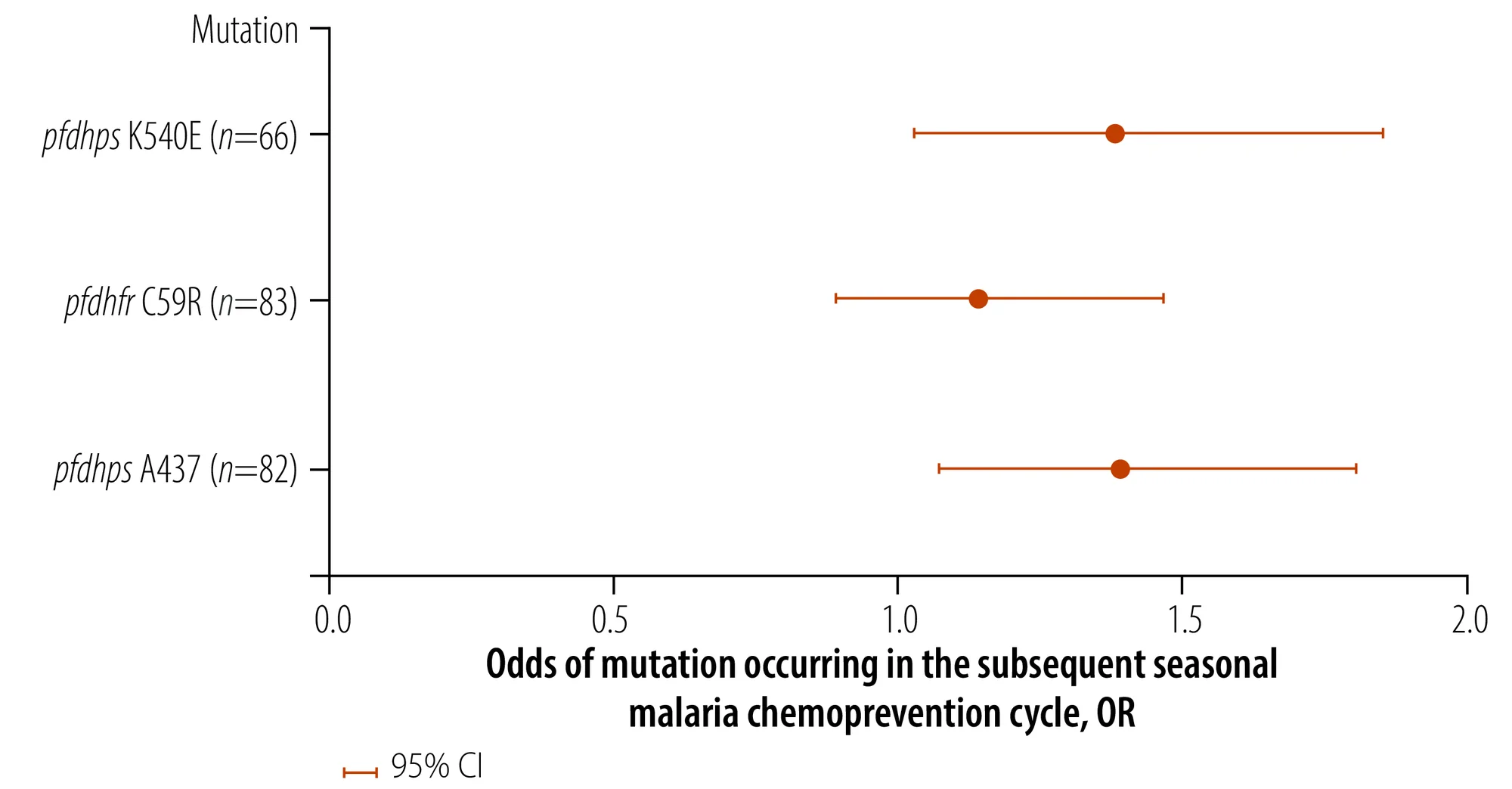
Odds of *Plasmodium falciparum* in infected children harbouring a *pfdhps* K540E, *pfdhfr* C59R or *pfdhps* A437G mutation in the subsequent seasonal malaria chemoprevention cycle, study of seasonal malaria chemoprevention and parasite drug resistance, Kenya, 2024

CI: confidence interval; OR: odds ratio; *P. falciparum: Plasmodium falciparum*.

Notes: in the regression analysis, the number of seasonal malaria chemoprevention cycles was treated as a continuous variable, ranging from 0 to 5. The odds can be interpreted as the relative change in the risk of infection by *Plasmodium falciparum* that harboured the specific mutation between one treatment cycle and the subsequent cycle. The odds ratios were not adjusted for any covariates.

## Discussion

Seasonal malaria chemoprevention is highly cost-effective for reducing morbidity and mortality from malaria among children. However, experience with the strategy in eastern and southern Africa is limited. In our study, seasonal malaria chemoprevention was successfully deployed in a unique community of pastoralists in northern Kenya and was associated with a significantly lower incidence of malaria episodes in the intervention cohort compared with the non-intervention cohort over the 5 months of the programme. The degree of protection observed in Turkana was lower than that reported in a study from neighbouring Uganda but was generally in agreement with the findings of randomized studies in West Africa and estimates from routine seasonal malaria chemoprevention.^6^^,^^30^^–^^32^ Nearly three quarters of malaria episodes in our intervention cohort occurred among children who received fewer than five cycles of chemoprevention, which suggests that the somewhat lower level of protection we observed was attributable to imperfect adherence to treatment. Although children in our study were in regular contact with community health promoters, less than three quarters were confirmed to have received all five cycles.

The remoteness and mobility of the study population were expected to present a challenge for attaining full coverage, and indeed more than a 10th of children missed appointments with the study team due to household movement. This proportion is higher than we recorded previously in cross-sectional studies and probably contributed to the suboptimal uptake of seasonal malaria chemoprevention.^33^

Parasite populations in East Africa have substantially higher levels, and a higher prevalence, of antifolate resistance than those in West Africa,^9^ which raises concerns about the efficacy of sulfadoxine–pyrimethamine and amodiaquine for seasonal malaria chemoprevention. Although sulfadoxine–pyrimethamine has remained effective for protecting pregnant women,^7^^,^^11^^,^^12^ it is possible that pre-existing immunity in the mother may play a role in boosting the drugs’ effects.^34^ Consequently, doubts linger about the effectiveness of sulfadoxine–pyrimethamine and amodiaquine for preventing malaria in young, non-immune children exposed to drug-resistant parasites. Nevertheless, several studies, including ours, confirm the high apparent efficacy of this combination in places where drug-resistant parasites predominate.^13^^,^^35^^–^^37^

To better understand the relationship between seasonal malaria chemoprevention and parasite resistance, previous studies have compared the prevalence of resistance alleles in *P. falciparum* genes before and after chemoprevention to determine whether the population prevalence of specific alleles may have increased.^6^^,^^38^^,^^39^ In our study, we focused on antifolate resistance alleles in clinical breakthrough infections that occurred during seasonal malaria chemoprevention to understand the relationship between the selection of sulfadoxine–pyrimethamine resistance genes in parasites and the effectiveness of malaria prevention. We found that the incidence of the *pfdhps* A437G mutation was significantly higher in incident *P. falciparum* infections among children receiving seasonal malaria chemoprevention than among infections in the comparison cohort. Moreover, the odds of harbouring mutant *pfdhps* 437 and *pfdhps* 540 increased with each chemoprevention cycle. A study in Burkina Faso demonstrated selection for two drug-resistance *dhfr* alleles (i.e. *dhfr* 59R and *dhfr* 108N) in prevalent infections during seasonal malaria chemoprevention.^40^ Although it is not known whether a small number of breakthrough infections makes a substantial contribution to the spread of sulfadoxine–pyrimethamine resistance, the observation that resistant parasites are more common in breakthrough infections is important because they may increase the risk of disease. 

The relationship between scaling up seasonal malaria chemoprevention with sulfadoxine–pyrimethamine and amodiaquine and changes in the prevalence of sulfadoxine–pyrimethamine-resistant parasites has been explored in countries where seasonal malaria chemoprevention is routinely implemented at scale. Typically, these studies measure changes in resistance alleles following chemoprevention using repeated, cross-sectional surveys of asymptomatic children or adults.^6^^,^^38^^,^^39^ Studies lasting 2 to 5 years have not found changes in antifolate resistance in parasite populations,^6^^,^^38^ but longer-term studies in Africa reported that antifolate resistance continued to expand over time.^39^ Although the relationship with seasonal malaria chemoprevention cannot be confirmed, it is the only substantial source of selective pressure from sulfadoxine–pyrimethamine. In East Africa, one study from Uganda reported no difference in the prevalence of antifolate resistance mutations in children within and outside areas where seasonal malaria chemoprevention was implemented.^13^ However, the baseline prevalence of resistance was very high, which left little room for an increase. A major weakness of these ecological studies is the absence of information on the use of seasonal malaria chemoprevention at the individual level, and of comparisons between individuals who are and are not eligible for seasonal malaria chemoprevention.

Our study has limitations that should be considered when weighing the evidence. This was not a randomized study. Instead, we selected comparison areas from which to draw the non-intervention villages to maximize their similarity to the intervention area at baseline, reduce confounding and, thereby, improve our inferences about the effect of the intervention. As children who had been treated with antimalarials in the previous two weeks were excluded from the next round of seasonal malaria chemoprevention, it is possible our weekly active monitoring of morbidity may have resulted in fewer children receiving chemoprevention than if less-sensitive passive case detection had been used. Moreover, the increased sensitivity of active case detection may have also led to malaria episodes being registered in children who might not have sought care at a health facility. However, both study cohorts would have been subject to this higher sensitivity and the estimated incidence ratios should not have been affected by bias. As we were able to characterize the three *P. falciparum* genomic positions of interest in less than half of all infections, our comparisons are imprecise, although the differences we found were significant. We did not evaluate amodiaquine resistance in infected children but the level of resistance was expected to be low in the region.^41^ Finally, it remains unclear whether the higher prevalence of sulfadoxine–pyrimethamine-resistant infections in children receiving seasonal malaria chemoprevention contributes to the broader spread of resistance at the population level. Even if resistant infections are transmitted by these children, the reduction in the overall number infected may offset this potential risk.

In conclusion, we found that the protection against malaria illness offered by seasonal malaria chemoprevention with sulfadoxine–pyrimethamine and amodiaquine was high despite the marked incidence of parasites with mutations in *dhfr* and *dhps* genes linked to sulfadoxine–pyrimethamine resistance. Consequently, expanding seasonal malaria chemoprevention to other regions that experience highly seasonal transmission would be worthwhile. However, given evidence that breakthrough infections by quintuple mutant parasites occur, routine monitoring of breakthrough infections in recipients of seasonal malaria chemoprevention should be established at health facilities to increase understanding of the contribution of resistance to morbidity. Moreover, information about local levels of resistance is important for predicting the parasite’s susceptibility to, and the overall efficacy of, chemoprevention.
